# Supplementary material for: Dietary Saccharomyces cerevisiae boulardii CNCM I-1079 Positively Affects Performance and Intestinal Ecosystem in Broilers during a Campylobacter jejuni Infection
Source: Microorganisms. 2019 Nov 21;7(12):596. doi: 10.3390/microorganisms7120596 (PMC6956328; doi:10.3390/microorganisms7120596)
Supplement: Supplementary file 1 [file microorganisms-07-00596-s001.zip › microorganisms-642415-submit-supplementary/Supplementary_materials/FIGURE_S2_B.pdf]

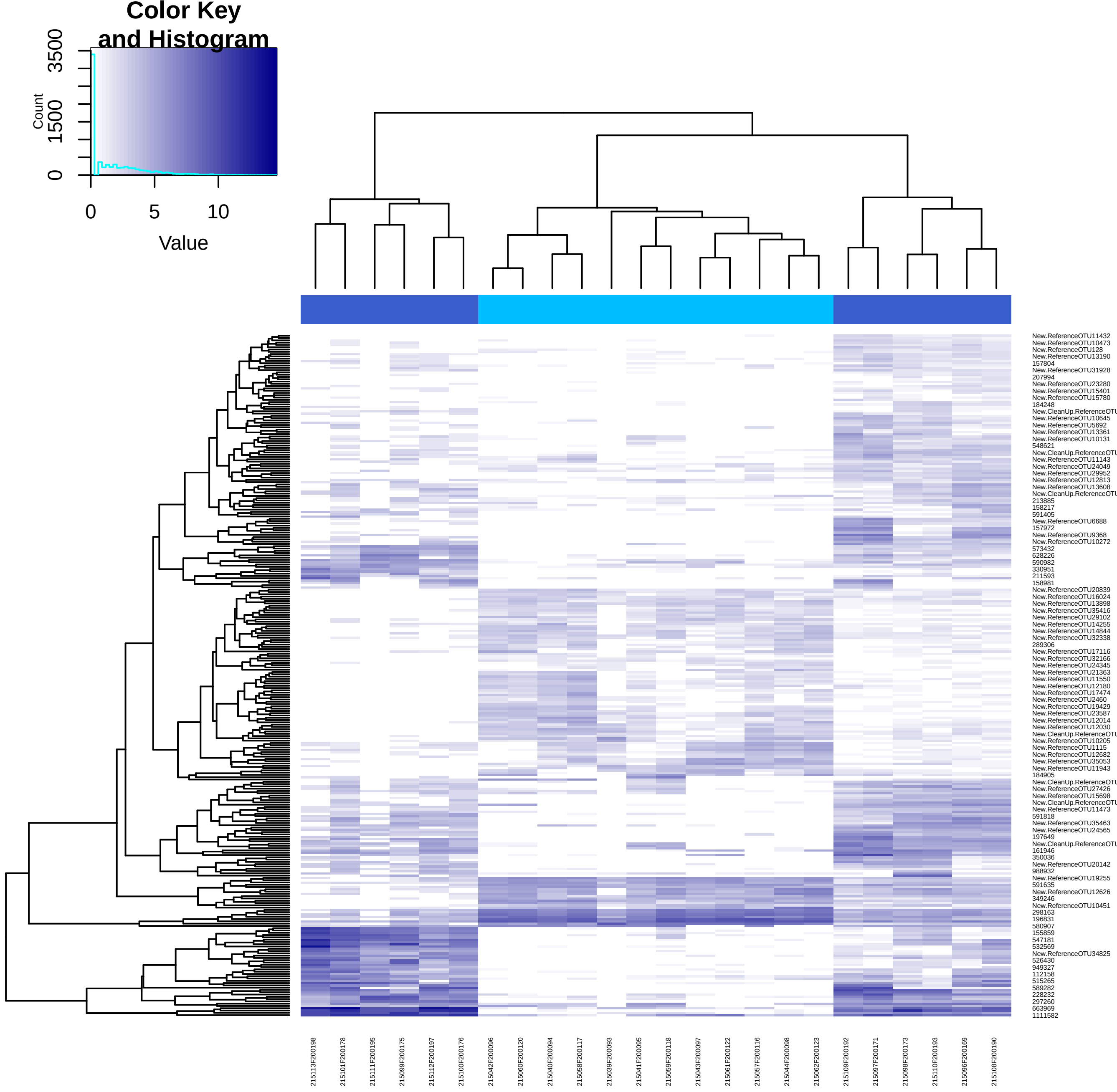

Color Key
and Histogram
Count
0
1500
3500
0
5
10
Value
215113F200198
215101F200178
215111F200195
215099F200175
215112F200197
215100F200176
215042F200096
215060F200120
215040F200094
215058F200117
215039F200093
215041F200095
215059F200118
215043F200097
215061F200122
215057F200116
215044F200098
215062F200123
215109F200192
215097F200171
215098F200173
215110F200193
215096F200169
215108F200190
New.ReferenceOTU11432
New.ReferenceOTU10473
New.ReferenceOTU128
New.ReferenceOTU13190
157804
New.ReferenceOTU31928
207994
New.ReferenceOTU23280
New.ReferenceOTU15401
New.ReferenceOTU15780
184248
New.CleanUp.ReferenceOTU
New.ReferenceOTU10645
New.ReferenceOTU5692
New.ReferenceOTU13361
New.ReferenceOTU10131
548621
New.CleanUp.ReferenceOTU
New.ReferenceOTU11143
New.ReferenceOTU24049
New.ReferenceOTU29952
New.ReferenceOTU12813
New.ReferenceOTU13608
New.CleanUp.ReferenceOTU
213885
158217
591405
New.ReferenceOTU6688
157972
New.ReferenceOTU9368
New.ReferenceOTU10272
573432
628226
590982
330951
211593
158981
New.ReferenceOTU20839
New.ReferenceOTU16024
New.ReferenceOTU13898
New.ReferenceOTU35416
New.ReferenceOTU29102
New.ReferenceOTU14255
New.ReferenceOTU14844
New.ReferenceOTU32338
289306
New.ReferenceOTU17116
New.ReferenceOTU32166
New.ReferenceOTU24345
New.ReferenceOTU21363
New.ReferenceOTU11550
New.ReferenceOTU12180
New.ReferenceOTU17474
New.ReferenceOTU2460
New.ReferenceOTU19429
New.ReferenceOTU23587
New.ReferenceOTU12014
New.ReferenceOTU12030
New.CleanUp.ReferenceOTU
New.ReferenceOTU10205
New.ReferenceOTU1115
New.ReferenceOTU12682
New.ReferenceOTU35053
New.ReferenceOTU11943
184905
New.CleanUp.ReferenceOTU
New.ReferenceOTU27426
New.ReferenceOTU15698
New.CleanUp.ReferenceOTU
New.ReferenceOTU11473
591818
New.ReferenceOTU35463
New.ReferenceOTU24565
197649
New.CleanUp.ReferenceOTU
161946
350036
New.ReferenceOTU20142
988932
New.ReferenceOTU19255
591635
New.ReferenceOTU12626
349246
New.ReferenceOTU10451
298163
196831
580907
155859
547181
532569
New.ReferenceOTU34825
526430
949327
112158
515265
589282
228232
297260
663969
1111582
