# Supplementary material for: Dietary Saccharomyces cerevisiae boulardii CNCM I-1079 Positively Affects Performance and Intestinal Ecosystem in Broilers during a Campylobacter jejuni Infection
Source: Microorganisms. 2019 Nov 21;7(12):596. doi: 10.3390/microorganisms7120596 (PMC6956328; doi:10.3390/microorganisms7120596)
Supplement: Supplementary file 1 [file microorganisms-07-00596-s001.zip › microorganisms-642415-submit-supplementary/Supplementary_materials/microorganisms-642415-submit-supplementary.docx]

**SUPPLEMENTARY DATA**

**Supplementary figures**


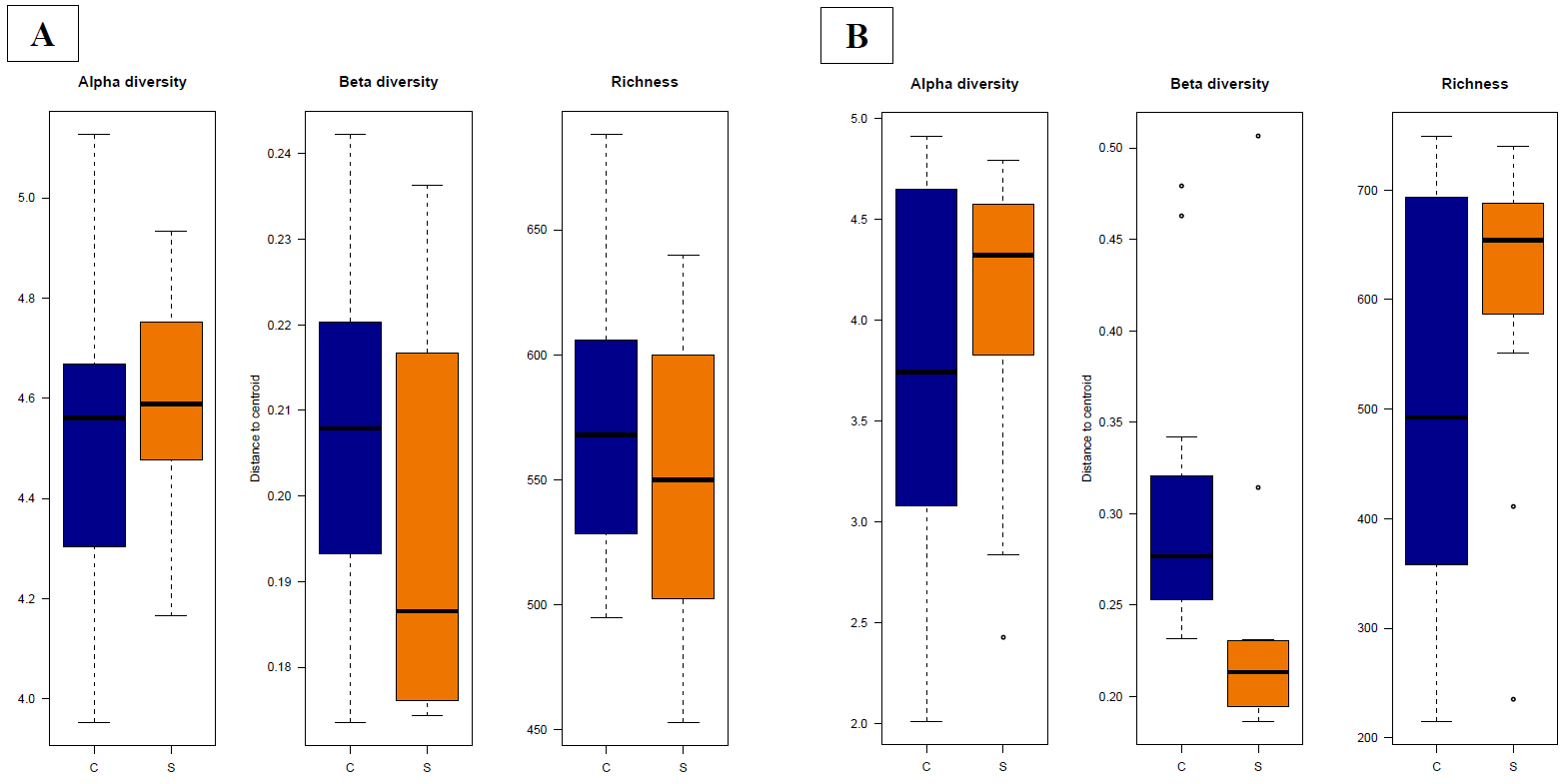


**Figure 1.** Box plots include only the samples obtained from fecal material. (A) Box plot graph representation of the alpha diversity (Shannon index), beta diversity (Whittaker's index) and richness (total number of OTUs present in each sample) using the rarefied OTU table for each group at d21; samples are colored by dietary treatment: C (basal control diet, blue) and S (basal diet supplemented with *Saccharomyces cerevisiae boulardii* CNCM I-1079; orange). (B) Box plot graph representation of the alpha diversity (Shannon index), beta diversity (Whittaker's index) and richness (total number of OTUs present in each sample) using the rarefied OTU table for each group at d40; samples are colored by dietary treatment: C (basal control diet, blue) and S (basal diet supplemented with *Saccharomyces cerevisiae boulardii* CNCM I-1079; orange).


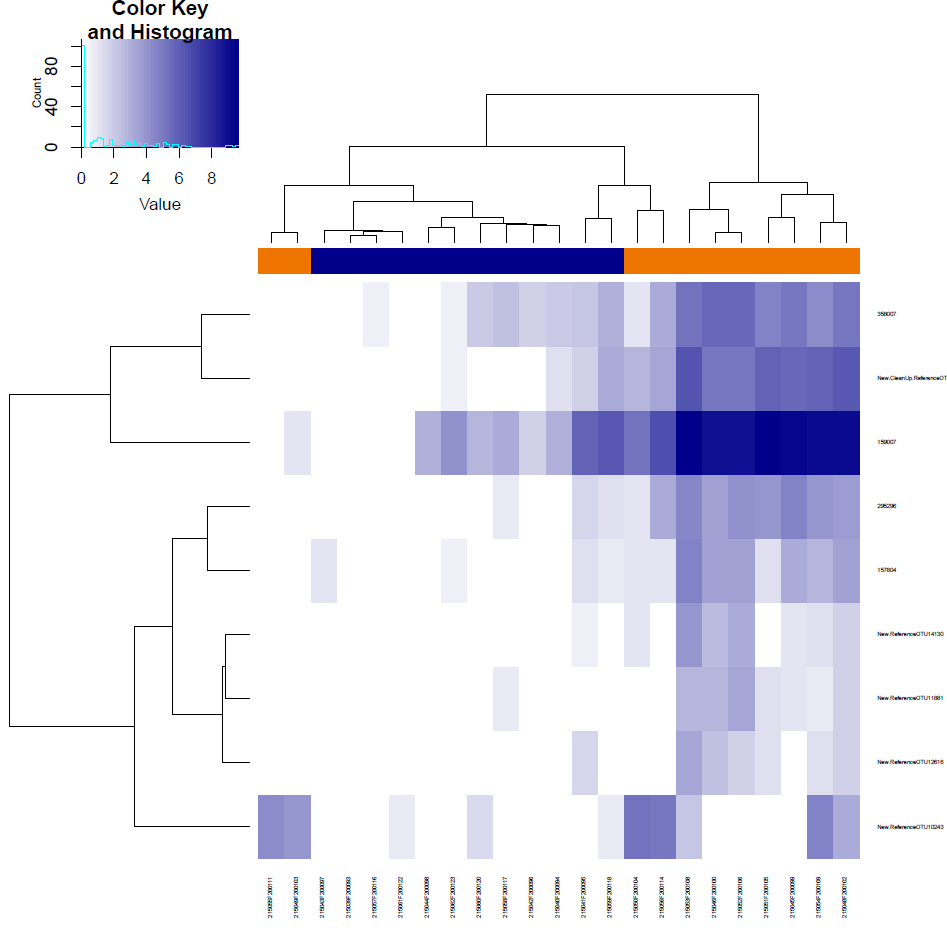


**A**


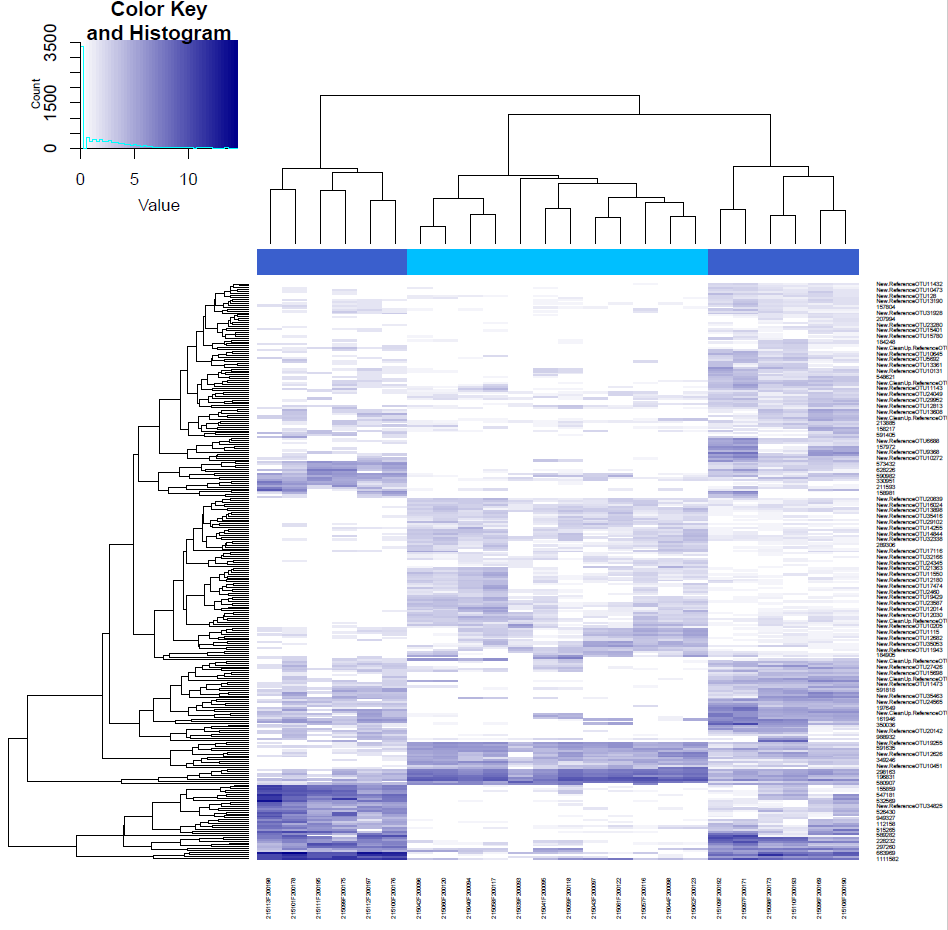


**B**


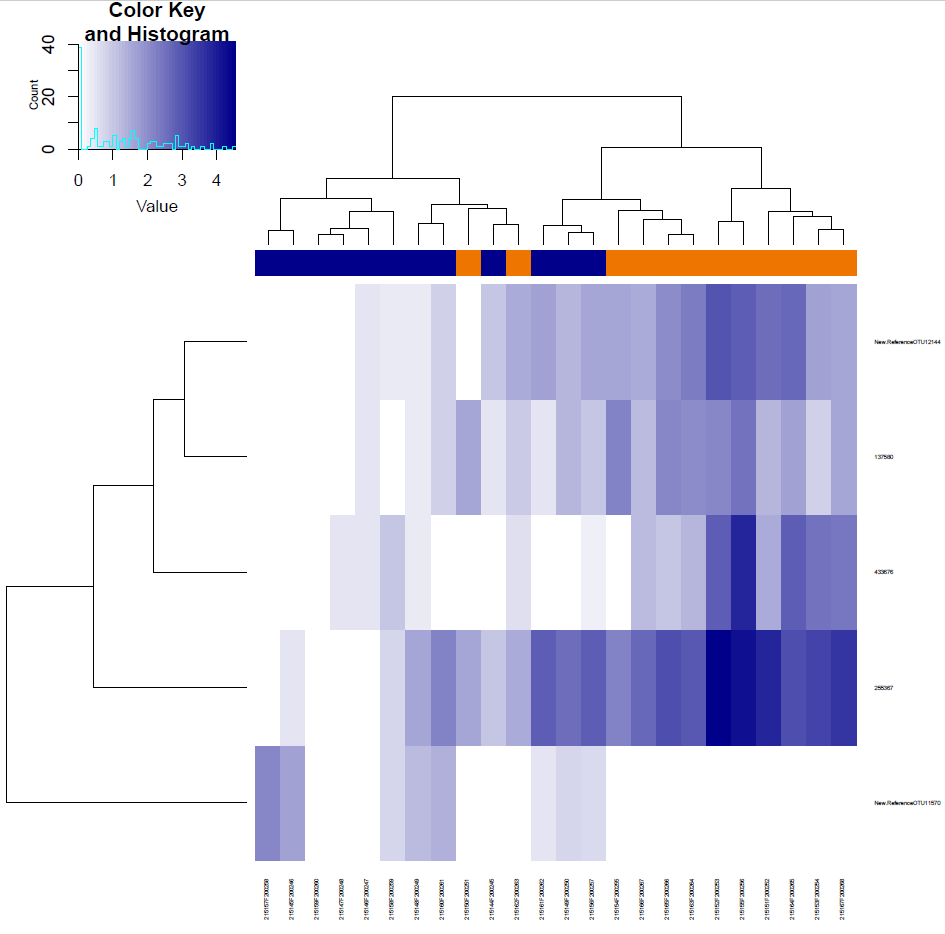


**C**

**Figure 2.** (A) Heat maps illustrating the abundances of differentially abundant (DA) OTUs expressed at d21 among the fecal samples of basal control diet (C; blue) and the basal diet supplemented with *Saccharomyces cerevisiae boulardii* CNCM I-1079 (S; orange) groups. (B) Heat maps illustrating the abundances of differentially abundant (DA) OTUs expressed at d21 (light blue) and d40 (dark blue) among the fecal samples of basal control diet group. (C) Heat maps illustrating the abundances of differentially abundant (DA) OTUs expressed at d40 among the cecal samples of basal control diet (C; blue) and the basal diet supplemented with *Saccharomyces cerevisiae boulardii* CNCM I-1079 (S; orange) groups.

**Table S1:** Ingredients and chemical composition of experimental feeds.

| **Ingredients (100g/kg)** |  | Starter |  | Grower-finisher |
| --- | --- | --- | --- | --- |
| Maize |  | 36.00 |  | 46.9 |
| Soybean meal |  | 27.00 |  | 25.00 |
| Corn gluten meal |  | 19.6 |  | -- |
| Corn gluten bran |  | -- |  | 15.1 |
| Maize distillers |  | 9.00 |  | -- |
| Rice bran |  | 4.20 |  | -- |
| Rice polishing |  | -- |  | 5.10 |
| Bran |  | -- |  | 4.00 |
| Soybean oil |  | 1.30 |  | 1.50 |
| Calcium carbonate |  | 1.29 |  | 1.37 |
| Dicalcium phosphate |  | 0.40 |  | -- |
| Sodium chloride |  | 0.30 |  | 0.30 |
| Sodium bicarbonate |  | 0.15 |  | -- |
| Premix^1^ |  | 0.76 |  | 0.73 |
|  |  |  |  |  |
| **Chemical Composition (%)** |  |  |  |  |
| Dry matter |  | 89.15 |  | 89.56 |
| Crude protein |  | 22.27 |  | 21.37 |
| Ether extract |  | 5.37 |  | 6.75 |
| Ash |  | 6.45 |  | 5.33 |
| NDF |  | 13.84 |  | 12.63 |
| ADF |  | 3.60 |  | 3.69 |
| Lignine |  | 0.48 |  | 0.49 |
| Lysine (calculated) |  | 1.16 |  | 0.79 |
| Methionine (calculated) |  | 0.50 |  | 0.35 |
| Calcium |  | 1.10 |  | 1.00 |
| Phosohorus |  | 0.52 |  | 0.44 |

^1^Mineral and vitamin Premix composition: Retinyl acetate 3a672a 10000 I.U., vitamin D3 E671 4500 I.U., vitamin E 3a700 50.00 mg, vitamin K 4.00 mg, vitamin B1 2.5 mg, vitamin B2 10.00 mg, vitamin B6 3a831 1.0 mg, vitamin B12 0.025 mg, biotin 3e800 0.20 mg, niacinamide 3a315 20.00 mg, folic acid 3a316 0.50 mg, calcium pantothenate 3a841 15.00 mg, ferrous carbonate 62.1 mg, ferric oxide 772 mg, potassium iodide 1.31 mg, copper sulphate pentahydrate 9.83 mg, manganese oxide 193.5 mg, zinc oxide 74,4 mg, DL-methionine 500 mg, phytase250 OUT, beta-glucanase 56 U, xilanase 216 U.

**Table S2:** Effect of *Saccharomyces cerevisiae* supplemented diets on cecal *Campylobacter* spp. and *Saccharomyces cerevisiae* population. Results were expressed as log_10_ cfu/g.

| **Cecal bacteria** |  | **Diet x Time** | | |  | **Overall** | |  |  | **Effects** | ***p*** |
| --- | --- | --- | --- | --- | --- | --- | --- | --- | --- | --- | --- |
|  |  | **Day 28** |  | **Day 40** |  |  |  |  |  |  |  |
|  |  |  |  |  |  |  |  |  |  |  |  |
| ***Campylobacter* spp.** |  |  |  |  |  |  |  |  |  |  |  |
| **C** |  | 8.70 |  | 8.66 |  | 8.68 |  |  |  | **Diet** | 0.3945 |
| **S** |  | 8.51 |  | 9.22 |  | 8.86 |  |  |  | **Time** | 0.1239 |
| **SEM** |  | 0.210 | | |  | 0.148 |  |  |  | **Diet x time** | 0.0863 |
|  |  |  |  |  |  |  |  |  |  |  |  |
| ***Saccharomyces* spp.** |  |  |  |  |  |  |  |  |  |  |  |
| **C** |  | 1.67 |  | 0.50 |  | 1.08 |  |  |  | **Diet** | <0.0001 |
| **S** |  | 5.04 |  | 4.99 |  | 5.01 |  |  |  | **Time** | 0.3105 |
| **SEM** |  | 0.587 | | |  | 0.415 |  |  |  | **Diet x time** | 0.3495 |

C: control diet

S: control diet supplemented with 1*10^9^ cfu/kg of *Saccharomyces cerevisiae boulardii* CNCM I-1079

SEM: standard error of the mean

**Table S3:** The OTU taxonomical assignments and OTU counts in each sample and time point of the whole dataset are showed.

**Table S4:** Study of global indicators of the gut ecosystem state, including evenness, dominance, divergences and rarity in our cohort.

**Table S5:** Cecal core microbiota in our cohort.

**Table S6:** Differentially abundant OTUs when comparing the fecal microbiota composition among the experimental groups at d21.

**Table S7:** Differentially abundant OTUs when comparing the fecal microbiota composition among the experimental groups at d40.

**Table S8:** Differentially abundant OTUs when comparing the fecal microbiota composition between d21 and d40 of the C (basal control diet) group.

**Table S9:** Differentially abundant OTUs when comparing the fecal microbiota composition between d21 and d40 of the S (basal diet supplemented with *Saccharomyces cerevisiae boulardii* CNCM I-1079) group.

**Table S10:** Differentially abundant OTUs when comparing the cecal microbiota composition of the experimental groups at d40.
