# Supplementary material for: Dietary Saccharomyces cerevisiae boulardii CNCM I-1079 Positively Affects Performance and Intestinal Ecosystem in Broilers during a Campylobacter jejuni Infection
Source: Microorganisms. 2019 Nov 21;7(12):596. doi: 10.3390/microorganisms7120596 (PMC6956328; doi:10.3390/microorganisms7120596)
Supplement: Supplementary file 1 [file microorganisms-07-00596-s001.zip › microorganisms-642415-submit-supplementary/Supplementary_materials/Table S1.docx]

**Table S1:** Ingredients and chemical composition of experimental feeds.

| **Ingredients (100g/kg)** |  | Starter |  | Grower-finisher |
| --- | --- | --- | --- | --- |
| Maize |  | 36.00 |  | 46.9 |
| Soybean meal |  | 27.00 |  | 25.00 |
| Corn gluten meal |  | 19.6 |  | -- |
| Corn gluten bran |  | -- |  | 15.1 |
| Maize distillers |  | 9.00 |  | -- |
| Rice bran |  | 4.20 |  | -- |
| Rice polishing |  | -- |  | 5.10 |
| Bran |  | -- |  | 4.00 |
| Soybean oil |  | 1.30 |  | 1.50 |
| Calcium carbonate |  | 1.29 |  | 1.37 |
| Dicalcium phosphate |  | 0.40 |  | -- |
| Sodium chloride |  | 0.30 |  | 0.30 |
| Sodium bicarbonate |  | 0.15 |  | -- |
| Premix^1^ |  | 0.76 |  | 0.73 |
|  |  |  |  |  |
| **Chemical Composition (%)** |  |  |  |  |
| Dry matter |  | 89.15 |  | 89.56 |
| Crude protein |  | 22.27 |  | 21.37 |
| Ether extract |  | 5.37 |  | 6.75 |
| Ash |  | 6.45 |  | 5.33 |
| NDF |  | 13.84 |  | 12.63 |
| ADF |  | 3.60 |  | 3.69 |
| Lignine |  | 0.48 |  | 0.49 |
| Lysine (calculated) |  | 1.16 |  | 0.79 |
| Methionine (calculated) |  | 0.50 |  | 0.35 |
| Calcium |  | 1.10 |  | 1.00 |
| Phosohorus |  | 0.52 |  | 0.44 |

^1^Mineral and vitamin Premix composition: Retinyl acetate 3a672a 10000 I.U., vitamin D3 E671 4500 I.U., vitamin E 3a700 50.00 mg, vitamin K 4.00 mg, vitamin B1 2.5 mg, vitamin B2 10.00 mg, vitamin B6 3a831 1.0 mg, vitamin B12 0.025 mg, biotin 3e800 0.20 mg, niacinamide 3a315 20.00 mg, folic acid 3a316 0.50 mg, calcium pantothenate 3a841 15.00 mg, ferrous carbonate 62.1 mg, ferric oxide 772 mg, potassium iodide 1.31 mg, copper sulphate pentahydrate 9.83 mg, manganese oxide 193.5 mg, zinc oxide 74,4 mg, DL-methionine 500 mg, phytase250 OUT, beta-glucanase 56 U, xilanase 216 U.
