# Supplementary material for: Dietary Saccharomyces cerevisiae boulardii CNCM I-1079 Positively Affects Performance and Intestinal Ecosystem in Broilers during a Campylobacter jejuni Infection
Source: Microorganisms. 2019 Nov 21;7(12):596. doi: 10.3390/microorganisms7120596 (PMC6956328; doi:10.3390/microorganisms7120596)
Supplement: Supplementary file 1 [file microorganisms-07-00596-s001.zip › microorganisms-642415-submit-supplementary/Supplementary_materials/Table S2.docx]

**Table S2:** Effect of *Saccharomyces cerevisiae* supplemented diets on cecal *Campylobacter* spp. and *Saccharomyces cerevisiae* population. Results were expressed as log_10_ cfu/g.

| **Cecal bacteria** |  | **Diet x Time** | | |  | **Overall** | |  |  | **Effects** | ***p*** |
| --- | --- | --- | --- | --- | --- | --- | --- | --- | --- | --- | --- |
|  |  | **Day 28** |  | **Day 40** |  |  |  |  |  |  |  |
|  |  |  |  |  |  |  |  |  |  |  |  |
| ***Campylobacter* spp.** |  |  |  |  |  |  |  |  |  |  |  |
| **C** |  | 8.70 |  | 8.66 |  | 8.68 |  |  |  | **Diet** | 0.3945 |
| **S** |  | 8.51 |  | 9.22 |  | 8.86 |  |  |  | **Time** | 0.1239 |
| **SEM** |  | 0.210 | | |  | 0.148 |  |  |  | **Diet x time** | 0.0863 |
|  |  |  |  |  |  |  |  |  |  |  |  |
| ***Saccharomyces* spp.** |  |  |  |  |  |  |  |  |  |  |  |
| **C** |  | 1.67 |  | 0.50 |  | 1.08 |  |  |  | **Diet** | <0.0001 |
| **S** |  | 5.04 |  | 4.99 |  | 5.01 |  |  |  | **Time** | 0.3105 |
| **SEM** |  | 0.587 | | |  | 0.415 |  |  |  | **Diet x time** | 0.3495 |

C: control diet

S: control diet supplemented with 1*10^9^ cfu/kg of *Saccharomyces cerevisiae boulardii* CNCM I-1079

SEM: standard error of the mean
