# Supplementary figures and images for: Dietary Saccharomyces cerevisiae boulardii CNCM I-1079 Positively Affects Performance and Intestinal Ecosystem in Broilers during a Campylobacter jejuni Infection
Source: Microorganisms. 2019 Nov 21;7(12):596. doi: 10.3390/microorganisms7120596 (PMC6956328; doi:10.3390/microorganisms7120596)

**A****Alpha diversity**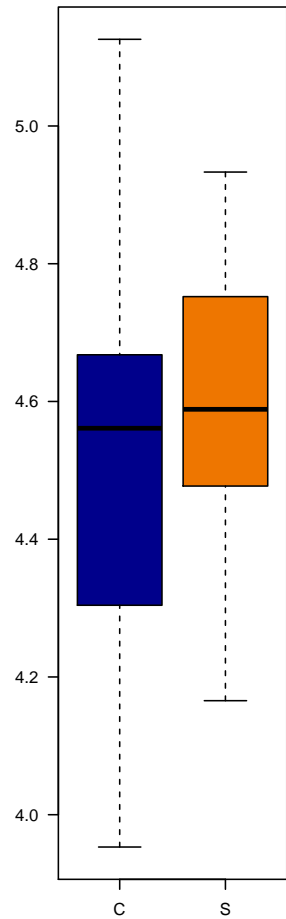**Beta diversity**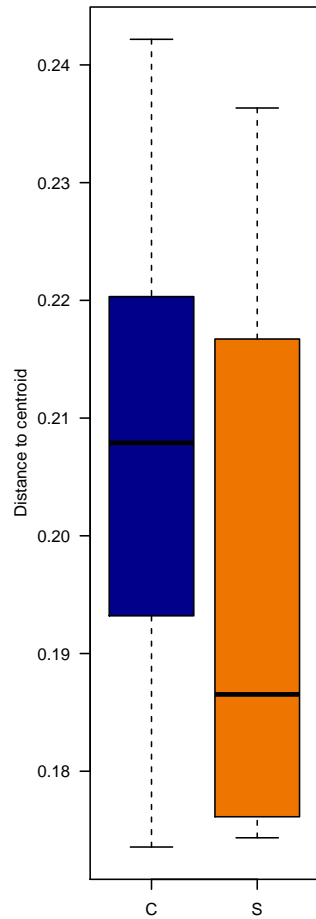**Richness**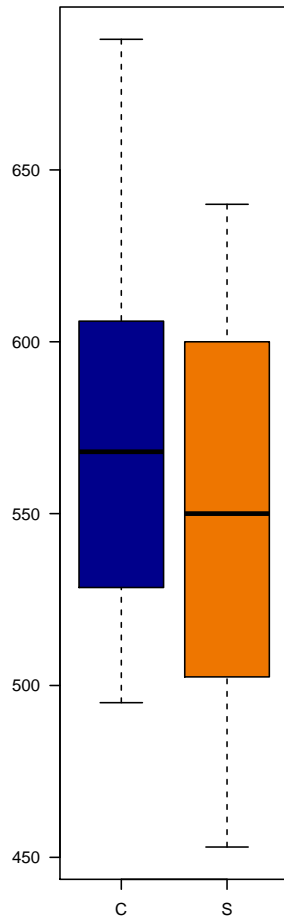**B****Alpha diversity**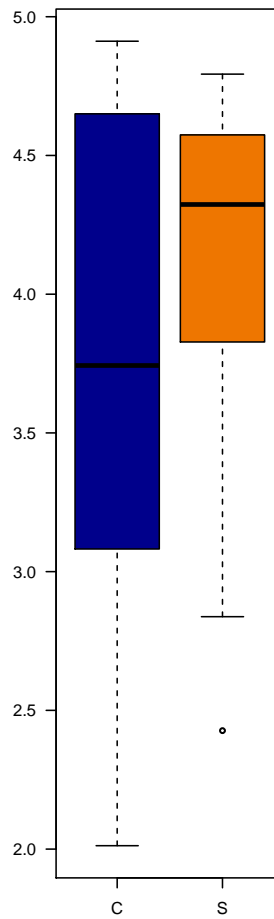**Beta diversity**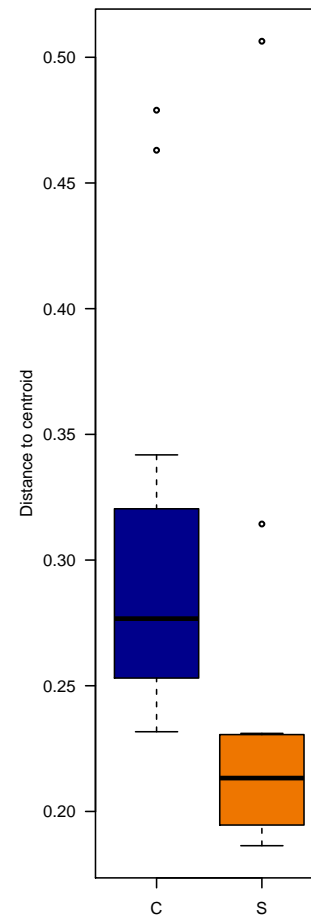**Richness**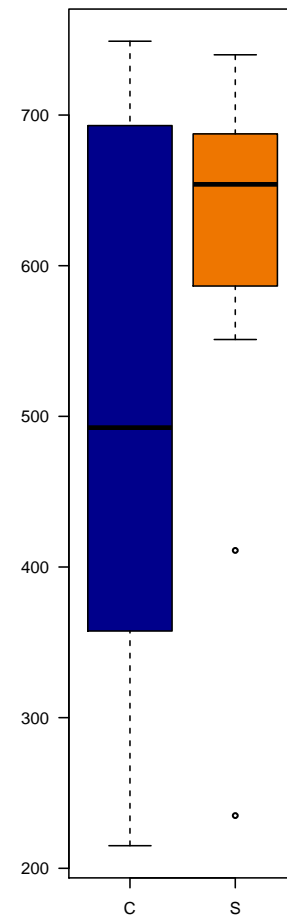

Supplement: Supplementary file 1 [file microorganisms-07-00596-s001.zip › microorganisms-642415-submit-supplementary/Supplementary_materials/FIGURE_S1.pdf]

# Color Key and Histogram

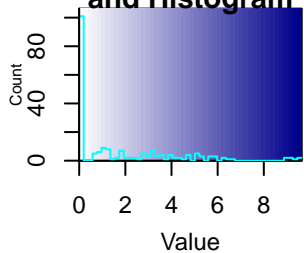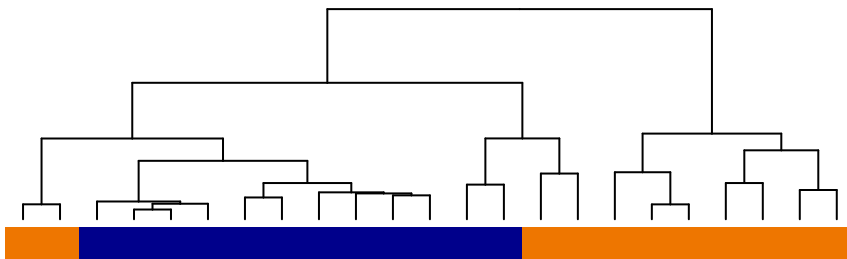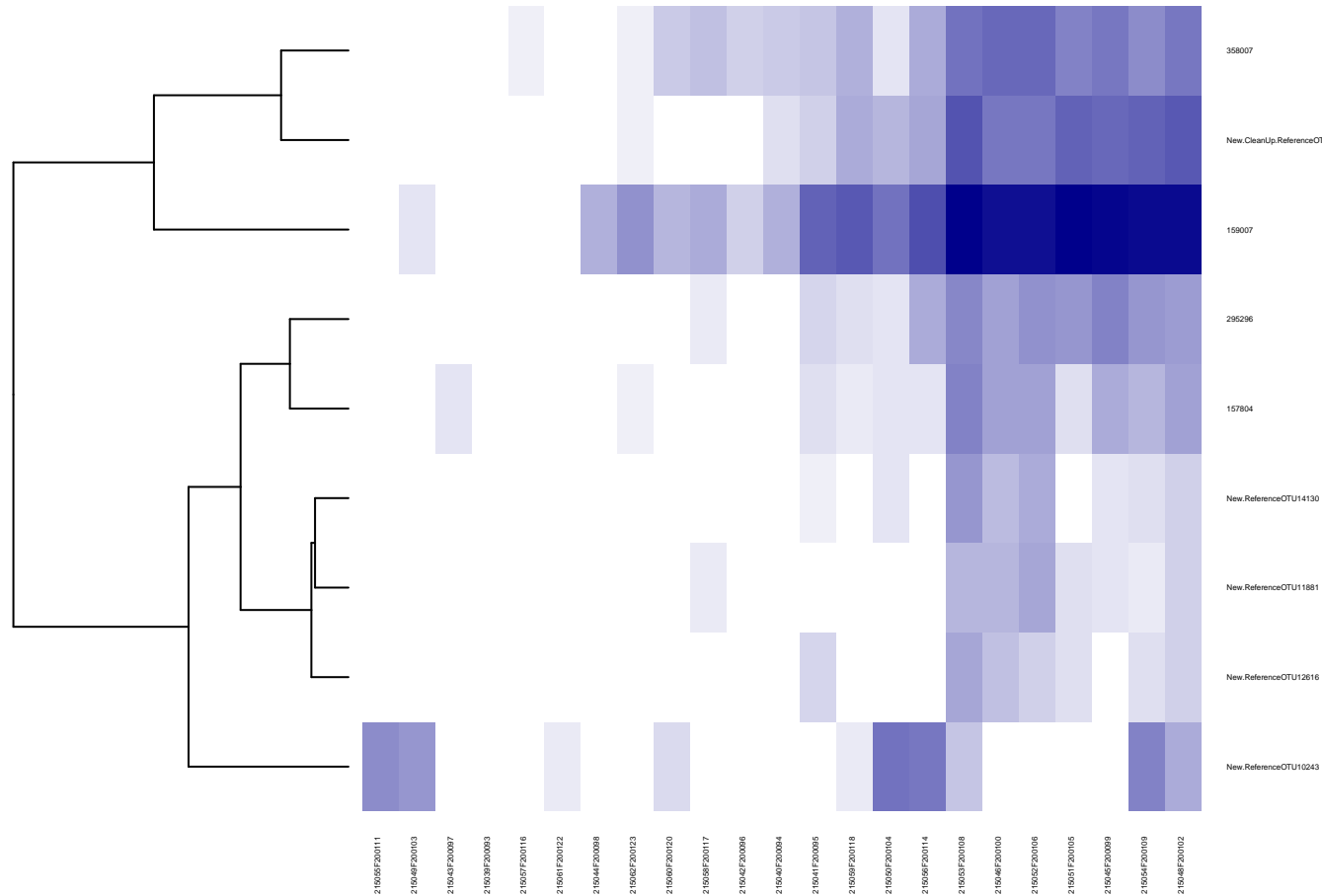

Supplement: Supplementary file 1 [file microorganisms-07-00596-s001.zip › microorganisms-642415-submit-supplementary/Supplementary_materials/FIGURE_S2_A.pdf]

# Color Key and Histogram

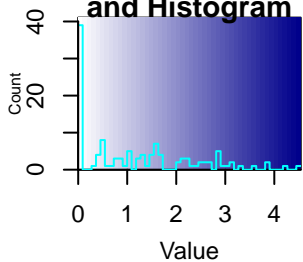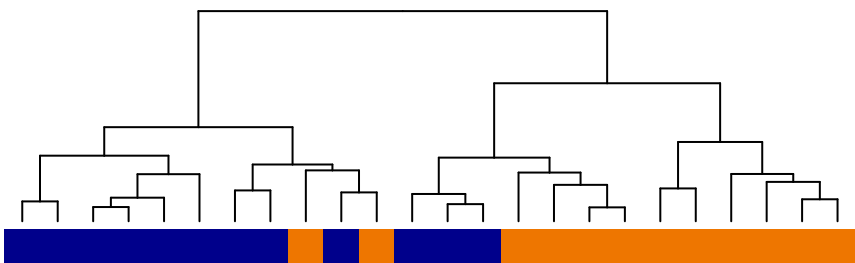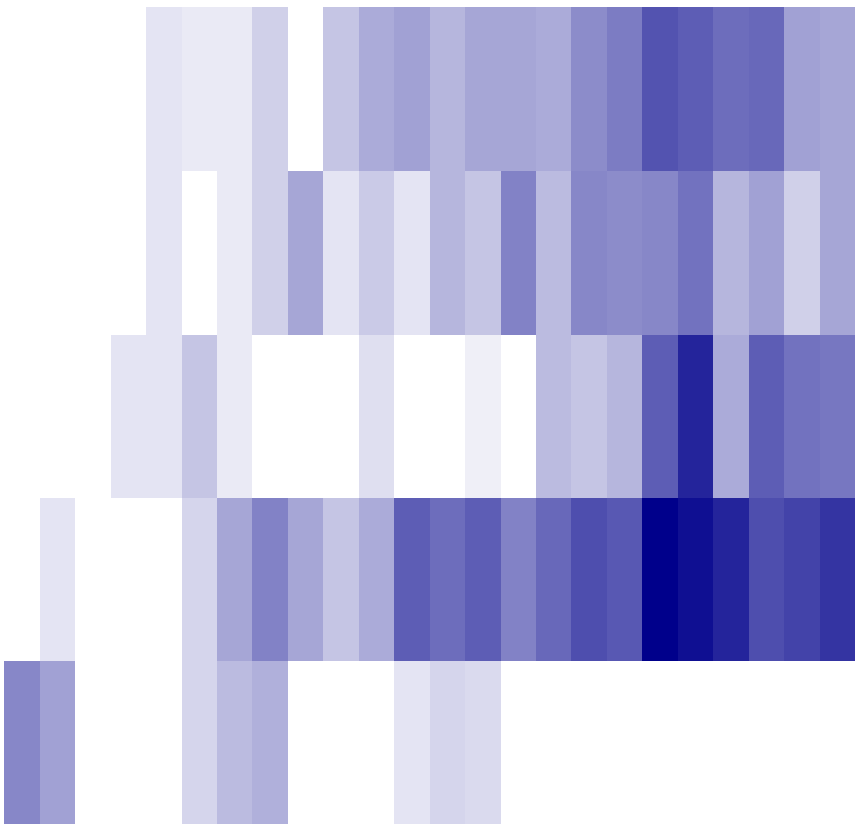

New Reference OTU12144

137580

433676

255367

New Reference OTU11570

Supplement: Supplementary file 1 [file microorganisms-07-00596-s001.zip › microorganisms-642415-submit-supplementary/Supplementary_materials/FIGURE_S2_C.pdf]
